# Effects of delayed NSAID administration after experimental eccentric contraction injury – A cellular and proteomics study

**DOI:** 10.1371/journal.pone.0172486

**Published:** 2017-02-28

**Authors:** Amy E. Bryant, Michael J. Aldape, Clifford R. Bayer, Eva J. Katahira, Laura Bond, Carrie D. Nicora, Thomas L. Fillmore, Therese R. W. Clauss, Thomas O. Metz, Bobbie-Jo Webb-Robertson, Dennis L. Stevens

**Affiliations:** 1 U.S. Department of Veterans Affairs, Office of Research and Development, Boise, ID, United States of America; 2 University of Washington School of Medicine, Seattle, WA, United States of America; 3 Northwest Nazarene University, Nampa, ID, United States of America; 4 Boise State University, Boise, ID, United States of America; 5 Pacific Northwest National Laboratory, Richland, WA, United States of America; West Virginia University School of Medicine, UNITED STATES

## Abstract

**Background:**

Acute muscle injuries are exceedingly common and non-steroidal anti-inflammatory drugs (NSAIDs) are widely consumed to reduce the associated inflammation, swelling and pain that peak 1–2 days post-injury. While prophylactic use or early administration of NSAIDs has been shown to delay muscle regeneration and contribute to loss of muscle strength after healing, little is known about the effects of delayed NSAID use. Further, NSAID use following non-penetrating injury has been associated with increased risk and severity of infection, including that due to group A streptococcus, though the mechanisms remain to be elucidated. The present study investigated the effects of delayed NSAID administration on muscle repair and sought mechanisms supporting an injury/NSAID/infection axis.

**Methods:**

A murine model of eccentric contraction (EC)-induced injury of the tibialis anterior muscle was used to profile the cellular and molecular changes induced by ketorolac tromethamine administered 47 hr post injury.

**Results:**

NSAID administration inhibited several important muscle regeneration processes and down-regulated multiple cytoprotective proteins known to inhibit the intrinsic pathway of programmed cell death. These activities were associated with increased caspase activity in injured muscles but were independent of any NSAID effect on macrophage influx or phenotype switching.

**Conclusions:**

These findings provide new molecular evidence supporting the notion that NSAIDs have a direct negative influence on muscle repair after acute strain injury in mice and thus add to renewed concern about the safety and benefits of NSAIDS in both children and adults, in those with progressive loss of muscle mass such as the elderly or patients with cancer or AIDS, and those at risk of secondary infection after trauma or surgery.

## Introduction

Non-steroidal anti-inflammatory drugs (NSAIDs) are cornerstones of pain management in homes and hospitals worldwide. These agents are consumed or prescribed for diverse conditions ranging from mild intermittent to chronic musculoskeletal pain, as well as for pain associated with cancer, AIDS, surgery and rehabilitation after placement of prosthetic devices. While NSAIDs have their place in these settings, they are not innocuous and can contribute to peptic ulcers, upper gastro-intestinal bleeding, renal disease and adverse cardiovascular events [1]. NSAID use has also been linked to poor wound healing and to increased risk of complications following surgery, including anastomotic failure following colorectal resection (reviewed in [2]).

NSAID use is widely advocated to manage the inflammation, pain and swelling associated with acute muscle strain injuries. Such injuries are exceedingly common among athletes, military personnel, and civilians of all ages. Within the first 24 to 48 hours after unaccustomed exercise or injury, a constellation of symptoms—referred to as delayed onset muscle soreness (DOMS)—develops that includes pain with muscle use, stiffness and swelling. Without treatment, these symptoms peak between 24 and 72 hours, and typically resolve within 5 to 7 days [3]. Yet, recent evidence suggests that the inflammatory response to injury is a necessary phase of soft tissue healing and its inhibition with NSAIDs can significantly delay muscle regeneration and decrease muscle strength after repair (reviewed in [4]). The deleterious NSAID effects on muscle repair have been attributed to their ability to block of cyclooxygenase-2 (COX-2)-derived prostaglandins which are known to stimulate muscle progenitor cell (satellite cell) responses to exercise [5–7]. These studies compared satellite cell activity in human volunteers or animal subjects when NSAIDs were administered *prior to* resistance training or to exercise-induced injury. In human studies, administration of non-selective NSAIDs (e.g., ibuprofen) blocked the exercise-induced increase in satellite cell number [7,8] and mixed muscle protein synthesis [9]. Similarly, animal studies demonstrate a negative effect of NSAIDs on satellite cell responses to exercise and on regeneration after injury [5,10]. Although NSAID use prior to exercise/injury is not a typical practice in the general population, these results clearly indicate that prophylactic use of NSAIDs, as is common among professional and elite athletes [4], exerts a negative effect on satellite cell activity.

In addition to their effects on muscle regeneration, recent clinical and epidemiological studies have suggested that NSAIDs may also predispose to severe bacterial infections including those due to *Clostridium difficile* [11,12], *Streptococcus pneumoniae* [13,14], *Staphylococcus aureus* [13,15], Gram negative organisms [15] and group A streptococcus (reviewed in [16]). Regarding the latter infection, a clinical entity termed “cryptogenic” or “spontaneous” group A streptococcal myonecrosis has been described in which patients initially present to their health care provider for increasing localized pain, typically at a site of non-penetrating soft tissue injury such as a muscle strain or bruise. Without an obvious portal of bacterial entry and in the absence of the classical cutaneous signs of developing infection, the correct diagnosis is missed or delayed. Instead, patients are prescribed NSAIDs and sent home, only to return 24–48 hrs later in multiple organ failure and intractable hypotension. Nearly 50% of streptococcal toxic shock syndrome (StrepTSS) patients with necrotizing soft tissue infection fall into this “cryptogenic” group [17]. Mortality is high (30–85%) and morbidity is extensive. By the time the systemic manifestations of StrepTSS are evident and the correct diagnosis of myonecrosis is made, emergent surgery—often including life-altering multiple limb amputations—is frequently required to ensure survival. Survivors are often left with profound cognitive impairments and endure prolonged hospitalization and rehabilitation at great emotional and financial expense.

Though a large body of clinical evidence supported a possible injury/NSAID/group A streptococcal axis, controversy remained. Opponents have argued that NSAID use merely masks the signs and symptoms of developing infection, thereby delaying diagnosis and treatment. Since no experimental studies had directly tested this possible association, we developed a murine model of exercise-induced muscle strain coupled with group A streptococcal bacteremia in which we investigated this notion [18]. We showed that NSAID administration 47 hrs post-injury significantly increased the trafficking of circulating group A streptococcus to the injured site [18]. In addition, using a murine model of established infection, we further demonstrated that three different non-selective NSAIDs each significantly increased disease severity and reduced the time to 100% mortality [19]. In total, this evidence suggested to us that NSAIDs directly contribute to poor outcomes in these infections.

As mentioned above, most studies examining the mechanisms by which NSAIDs affect muscle regeneration used models in which NSAIDs were given *prior to* resistance training or exercise-induced injury. Few studies investigated NSAID effects when given later–at times analogous to the peak of perceived muscle soreness in humans. Thus the present study utilized flow cytometric analysis of infiltrating inflammatory cells and an unbiased proteomics approach to investigate the cellular and protein changes in murine tibialis anterior (TA) muscle after eccentric contraction (EC)-induced muscle injury in the presence and absence of ketorolac tromethamine–a non-selective NSAID—given 47 hrs after muscle injury.

Findings document a predominantly inhibitory effect of ketorolac on muscle metabolism and biogenesis that was independent of any significant change in the numbers of infiltrating inflammatory cells or their functional phenotypes. Instead, results suggest that NSAID use at this time significantly down-regulates several anti-apoptosis proteins that inhibit the extrinsic pathway of programmed cell death. Such down-regulation of pro-survival proteins was accompanied by increased caspase activity in the muscle. By promoting a pro-apoptosis phenotype in regenerating muscles, NSAIDs could expand the nidus of injury, and thereby increase muscle susceptibility to post-injury infection. This activity could also contribute to the known ability of NSAIDs to delay muscle regeneration and reduce muscle strength post-healing. These findings warrant further studies in humans and if confirmed, this new knowledge may shift the current paradigm of pain management in multiple clinical settings.

## Methods

### Ethical approval

Studies involving animal subjects were approved by the Institutional Animal Care and Use Committee (IACUC) U.S. Department of Veterans Affairs Medical Center, Boise, ID and adhered to guidelines specified by the Veterans Health Administration (handbook 1200.07), the U.S. Public Health Service and the National Institutes of Health. Work was conducted in the Boise VA Medical Center’s Association for Assessment and Accreditation of Laboratory Animal Care (AAALAC)-accredited Veterinary Medical Unit. Animals were housed in groups of 5–10 in standard large rodent cages and allowed food and drink *ad libitum*. All investigators understand this journal’s ethical principles regarding animal subjects in research and confirm that our work complies with these principles.

### Animal model

This model has been previously described in detail [18]. In brief, adult Swiss Webster outbred mice (female, 20–25 g, Taconic Laboratories; N = 9/group) were anesthetized with 2% inhaled isoflurane in oxygen and positioned in the temperature-controlled exercise apparatus (modified model 360B, Aurora Scientific, Ontario, Canada) that permits control of both ankle dorsiflexion torque and muscle length. Anesthesia was maintained throughout the experimental procedure via delivery of 2% isoflurane via nose cone and breathing rates were carefully monitored. Adequacy of anesthesia was defined as no withdrawal response to toe-pinch.

The tibialis anterior (TA) muscle was stimulated electrically (10–12 volts, 100–150 Hz for 400 ms) by sterile 28-gauge needle electrodes placed subcutaneously ~2 mm apart near the right peroneal nerve just lateral to the midline and distal to the knee joint. Beginning at 200 ms after nerve stimulation and at maximal muscle contraction, the foot was rotated distally 78 degrees. At the end of the stimulation period, the foot was returned to the starting position. This exercise regimen was repeated every 30 seconds 50 times. Prior to, and immediately after exercise, the maximal mean isometric torque (MIT) generated by two isometric contractions (i.e., the foot held motionless) was recorded in each animal. Only animals displaying a post-EC reduction in MIT of >40% were included for study since we [18] and others [20] have previously shown that this level of functional impairment constitutes a moderate muscle injury. The contralateral leg of each animal was not exercised and served as control. After exercise, a single dose of buprenorphine (0.1 mg/kg in 0.5 mL sterile saline) was administered to minimize post-exercise distress and to replenish fluids lost during the 1 hr exercise regimen. This opioid analgesic agent is active at the central nervous system level; the single dose used is 20-fold less than that shown to have modest transient anti-inflammatory activity in the rat (2 mg/kg q 12 hr) [21].

At 47 hrs after EC, animals were treated with ketorolac tromethamine (trade name, Toradol; 7.5 mg/kg by IP injection) or its vehicle (saline). Ketorolac is a prescription non-selective NSAID commonly used in clinics and emergency room settings for short-term management of moderately severe, acute muscle pain in adults. It was chosen for these studies because ***1)*** it is of the same chemical subclass as ibuprofen (propionic acid) with similar COX1/COX2 inhibitory-50 [IC_50_] ratios (.35 and .5, respectively) [22] and can be given parenterally to experimental animals; ***2)*** in our previously published experimental studies in mice, ketorolac significantly increased trafficking of circulating group A streptococcus to sites of EC-induced muscle injury [18] and ***3)*** in established group A streptococcal myonecrosis in mice, ketorolac was equivalent to ibuprofen and indomethacin in the ability to significantly increase severity of infection and decrease the time to 100% mortality [19]. The time of NSAID administration was chosen for several reasons: ***1)*** our data (below) and those from Lieber’s group [23,24] have shown that muscle force-generating capacity remains significantly reduced at 48 hrs post injury and that this dysfunction is associated with marked architectural and inflammatory changes and in myoregulatory gene expression [23,24]; ***2)*** this time post-injury is related to the peak soreness in humans after unaccustomed exercise or muscle injury and when NSAID use is prevalent and ***3)*** studies by us (presented here) and by Arnold et al [25] have shown that by this time after experimental strain injury, polymorphonuclear leukocyte (PMNL) influx has waned and infiltrated pro-inflammatory macrophages have begun conversion to an anti-inflammatory, pro-resolution phenotype; thus any NSAID effect that might be observed would not be attributable to its ability to limit the tissue leukocytic inflammatory response.

Seven hours after NSAID or vehicle administration (54 hr post-injury), mice were anesthetized as described above, heparinized blood specimens obtained by retro-orbital puncture and, while still under anesthesia, the animals were sacrificed by cervical dislocation. The TA muscles from the left and right legs were harvested and flash frozen for proteome analysis. The timing of muscle harvest was based on our previously published work demonstrating a significant NSAID-induced increase in trafficking of group A streptococcus to the TA muscle at this time post-injury [18]. Plasma samples were frozen at -70C. In separate studies, animals were exercised as described above and treated with NSAID or saline at 47 hrs post injury. At selected times thereafter as indicated in the figure legends, TA muscles were harvested post-mortem and were either flash frozen for quantitative RT-PCR analysis for genes of interest, fixed in neutral buffered formalin for routine histopathology or processed for leukocyte isolation or caspase activity assay as described below.

### Proteomic analyses

The accurate mass and time (AMT) tag approach [26] was used to generate quantitative proteomics data for muscle tissues from the above described animals (see text in S1 Supporting Information: Additional Methodology for Proteomics Analysis for full experimental details). Comprehensive shotgun proteomics analyses were first performed to generate an AMT tag database of identified mouse peptides/proteins. This database was then used to identify injury- and NSAID-associated quantitative and qualitative differences in protein expression. Specifically, injured and non-injured TA muscles from each animal treated with or without NSAIDs at 47 h after injury (9 mice/group) as described above were harvested at 7 h after NSAID (or vehicle) administration, flash frozen and sent on dry ice to Pacific Northwest National Laboratory. Tissues from injured and non-injured TA muscles from all treatment groups were individually homogenized, digested using trypsin, and aliquots from each digest were pooled and subjected to offline fractionation [27]. Each fraction was then analyzed by nano capillary LC-MS/MS, and the identified peptides were used to populate the mouse muscle peptide/protein AMT tag database. Peptides were identified with a false discovery rate <0.1% and with a mass measurement error of +/- 4ppm.

The resultant comprehensive protein database was then used as a ‘look up table’ for the subsequent treatment group-specific quantitative proteomics analyses, which involved analysis of the individual tissue protein digests using the same LC-MS instrument platform, followed by data analysis using the AMT tag approach. Briefly, the observed masses and normalized elution times (NETs) of peptides detected in the individual samples were matched against the entries in the AMT tag database using the PRISM Data Analysis system [28], which is a series of software tools developed in-house (e.g. Decon2LS [29] and VIPER [30]) and freely available at omics.pnl.gov/software. Individual steps in this data processing approach are reviewed in [26]. The peptides identified from this matching process and the associated integrated LC-MS peak areas were retained as a matrix for subsequent data analysis.

LC-MS log transformed peak intensity (i.e., abundance) data was processed through appropriate quality control (QC) filters. One mouse sample did not meet adequate quality metrics associated with correlation and missing values and was removed from subsequent analyses [31]. Following QC, the dataset was appropriately normalized to reduce systematic effects of the analytical process, such as variation in total sample protein. The log transformed normalized peptide abundance dataset was appropriately filtered to remove peptides with inadequate data for statistical analysis. All proteins having 1 or more peptides passing the above-mentioned filters were assessed.

Each protein that met both the criteria of sample-level “Coverage” [Number of paired injured and control muscles ≥ 50% or (N_(injured)_ or N_(control)_) ≥75%] and of sample-level “Difference” [|Fold Change| ≥1.25 or |N_(injured)_—N_(control)_| ≥ 50%) were subjected to statistical analysis to identify those proteins that were significantly affected by injury or by NSAID. Proteins were analyzed for quantitative effects associated with injury or with NSAID administration using a standard T-test and for qualitative effects using G-test methodology [32]. The T-test indicated proteins that are present in both treatment groups that have a quantitative change in expression, i.e., up- or down-expression, and G-test indicated proteins that are present in one group and absent from another for identification of qualitative changes. Protein quantitative measures were computed using BP-Quant [33] and standard averaging methods [34] and statistical analysis followed the same T-test and G-test strategies.

### Functional analysis

Diseases and functions associated with injury +/- NSAID administration were identified in part using QIAGEN's Ingenuity Pathway Analysis (IPA, version 9.0, QIAGEN Redwood City, www.qiagen.com). Briefly, a dataset of significantly altered proteins containing the protein identifier, the P value, and the fold-change was uploaded into IPA and a core analysis was performed. For proteins that were significant by G-test (and for which a fold-change is not relevant), values of 2 or -2 were assigned to indicate those that were qualitatively increased or decreased with treatment, respectively, compared to non-treated controls.

In addition, initial protein functional categorization was performed by GeneOntology’s PANTHER classification system (www.pantherdb.org). Primary function was further refined based on descriptions provided by the NCBI Protein database and the available literature. Here, the functional categories were based on the updated Clusters of Orthologous Groups (COG) of proteins described by Tatusov et al [35] with some modifications. For multi-functional proteins, the primary category was assigned by considering a role in skeletal muscle structure, function and biogenesis.

### Gene expression analyses

The TA muscles from both exercised and the contra-lateral, non-exercised legs of saline- or NSAID-treated animals (4-12/group) were harvested at 4 or 7 hrs post-treatment (51 and 54 hrs post-injury) and immediately flash-frozen in liquid nitrogen for subsequent analysis of gene expression by real time quantitative reverse transcriptase PCR (qRT-PCR). Frozen muscles, suspended in TriReagent and 4-bromoanisole (Molecular Research Center Inc, Cincinnati, OH), were disrupted in a TissueLyser II (Qiagen, Valencia, CA). Total RNA was extracted from the resulting homogenate using an RNeasy RNA isolation kit (Qiagen), then treated with TURBO DNA-free (Ambion, Grand Island, NY) to remove contaminating DNA. RNA quantity was determined by Qubit (Invitrogen, Grand Island, NY) analysis and quality was assessed by TapeStation (Agilent, Santa Clara, CA) analysis. cDNA was synthesized from 2 micrograms of isolated total RNA using MMLV reverse transcriptase (New England Biolabs, Ipswich, MA) then diluted 1:10. Following manufacturer’s protocol, 1ul of the diluted cDNA was added to 96 well reaction plates (MicroAmp™ Fast Optical plates; Applied Biosystems) containing 1X SYBR Green/Rox PCR master mix (Qiagen) and 0.4uM RT^2^ qPCR Primer Assay mix for murine genes of interest (Qiagen, Valencia, CA). qRT-PCR was performed on either an ABI 7500 Fast PCR (Applied Biosystems) or Realplex 2 (Eppendorf) machine using the following parameters: 95°C for 10 min; 40 cycles of 95°C for 15 sec and 60°C for 1 min. A dissociation (melting) curve was run immediately after the PCR program to verify product integrity. The threshold cycle (Ct) for each transcript and change in threshold cycle (ΔCt) for the gene of interest/GAPDH pair of transcripts were monitored for each amplification reaction and the data was analyzed using the 2^-ΔΔCt^ method [36] to calculate fold change between the exercised and non-exercised muscles. Statistical differences were determined by inspection of 95% confidence intervals (CI) of the log_2_-transformed fold-change values (ΔΔC_t_; non-coverage of 0 by the CI indicates statistical difference between treatment and control at p < 0.05).

### Leukocyte isolation

To assess the dynamics and phenotypic characteristics of infiltrating cellular response after injury, TA muscles from 3 animals/group were removed and digested following the methods of Arnold et al [25] with some modifications. Muscles were rinsed in DPBS then digested twice in DMEM containing 0.2% collagenase B and 0.2% trypsin at 37°C for 1 hr. Resultant material was filtered with a 70 μm cell strainer. Isolated cells were recovered by centrifugation and suspended in PBS pH 7.2, 0.5% BSA, and 2mM EDTA and passed through a 30 μm MACS pre-separation filter (Miltenyi Biotec, Auburn, CA). After treatment with an FcR blocking reagent (Miltenyi Biotec) to prevent nonspecific binding, CD11b+ cells were isolated by positive selection using magnetically labeled microbeads conjugated to anti-murine CD11b antibody (rat IgG2b; clone M1/70.15.11.5; Miltenyi Biotec) and selected by magnetic column according to the manufacturer’s instructions. Murine monocytes/macrophages (MO/MP) express abundant, high affinity surface CD11b. In contrast, resting granulocytes (including eosinophils) and NK cells express fewer, lower affinity CD11b molecules; upon activation, a functional up-regulation (i.e., increased affinity) as well as increased CD11b expression is observed. Total cell number was assessed by direct hemocytometer counting and viability was >98% by trypan blue exclusion.

A portion of the final cell suspension was fixed to a microscope slide by cytospin, stained with Wright’s stain, and a manual differential performed by a blinded animal pathologist at the Caine Veterinary Teaching Hospital, Caldwell, Idaho. To determine the phenotype of the isolated MO/MP, cells were incubated with either PE-Ly-6C (Miltenyi Biotec), FITC-F4/80 (eBioscience), or isotype control antibodies (eBioscience) at 4°C for 10 min. Cells were washed once and resuspended in 0.5 mLs PBS with 0.5% BSA and 2 mM EDTA. Analysis was immediately performed on an Epics XL flow cytometer with the monocyte gate drawn based on size and granularity. An average of 10,000 events was analyzed per sample. MO/MPs in the contra-lateral, non-exercised TA muscle were similarly isolated in parallel, however too few cells were recovered for subsequent analysis. Statistical differences were assessed using the Mann-Whitney test, based on an exact distribution [37,38], as an alternative to the parametric t test.

### Caspase activity assay

TA muscles were harvested from injured and non-injured legs 7 hrs after NSAID or vehicle treatment (i.e., 54 hrs post-injury). Each TA muscle (4/group) was flash frozen and pulverized by Qiagen Tissuelyzer in 250 uL of extraction buffer (25 mM HEPES pH 7.5, 0.1% Triton X-100, 5 mM MgCl_2_, 2 mM DTT, 74 uM antipain, 0.15 uM aprotinin, 1.3 mM EDTA, 20 mM leupeptin and 15 uM pepstatin). Digests were clarified by centrifugation (15,000xg, 20 min) and protein concentration determined by bicinchoninic acid assay (BCA) assay (Pierce). Combined activities of caspases 3 and 7 in the digests were measured by commercial ELISA (Promega) which utilizes a profluorescent DEVD peptide-rhodamine 110 substrate [(Z-DEVD)2-R110]. Data are reported as Relative Luminescence Units (RLU)/mg protein normalized to that of the non-injured control. Statistically significant differences were determined by ANOVA using a mixed model with random effect of animal within treatment. The level of significance was set at *P* < .05.

## Results

### Eccentric contraction model of muscle injury

To investigate the effects of injury with and without NSAID treatment on the regenerating skeletal muscle proteome and cellular inflammatory response, we utilized our established murine model of eccentric contraction (EC)-induced muscle injury [18]. This model was chosen since 1) EC injury is more relevant to the human condition than is freeze- or toxin-induced injury, 2) the TA muscle largely consists of Type II (fast twitch) fibers [39] thereby obviating the confounding influence of multiple fiber types in analysis; 3) fast-twitch muscles are considered more susceptible to EC-induced injury [40] and 4) because NSAID consumption for pain and swelling is highly associated with such injury [40].

**Fig 1:**
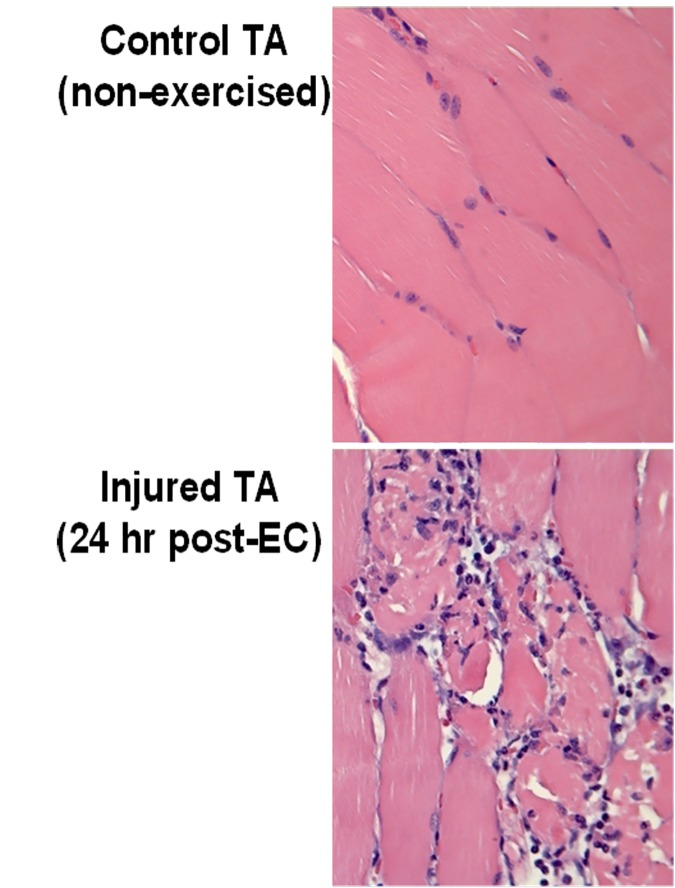
Experimental muscle injury disrupts myofiber architecture and causes marked influx of inflammatory cells. Mice underwent the eccentric contraction (EC) exercise regimen as described in Methods. At 24 hr post-EC, animals were euthanized and both left and right tibialis anterior (TA) muscles were harvested post-mortem for routine histopathology. One representative animal of 3 is depicted. The exercised muscle, but not the control TA, displays destruction of normal muscle architecture and a marked inflammatory cell influx. These pathologies, and the corresponding reduction in muscle force, are accepted criteria that define muscle injury.

**Fig 2:**
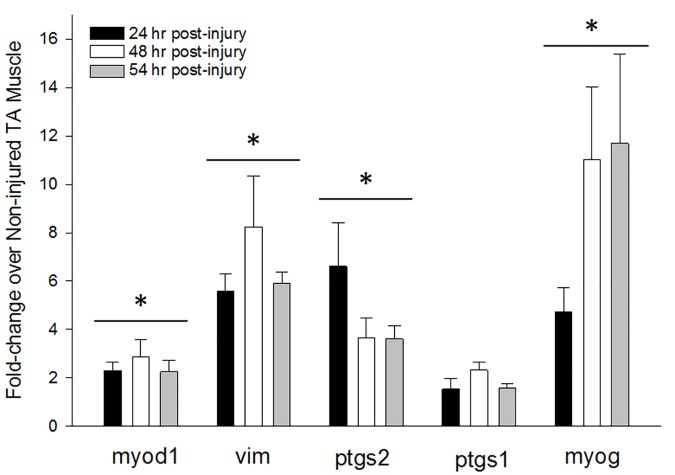
Experimental eccentric contraction injury stimulates physiological responses characteristic of muscle regeneration. Mice (4-12/group) underwent the EC regimen described above. Non-injured and injured TA muscles were harvested at the indicated times post-injury, flash-frozen and used to evaluate the relative expression of genes involved in muscle regeneration by qRT-PCR as described in Methods. Data are given as the mean fold-change in gene expression relative to the non-injured muscle ± standard error of the mean (SEM). Calculation of the 95% confidence interval using the log_2_-transformed fold-change values revealed that all genes were significantly increased at each time point relative to the non-injured control as indicated by the grouped asterisks, except the 24h *ptgs1* expression. No corresponding proteomics analyses were conducted at 24 or 48 hrs; at 54 hrs post-injury the only corresponding protein that was significantly altered by injury was vimentin (Table in S1 Table: Proteins Significantly Altered by Injury; 1.745-fold increase; *p* = .0236).

Repeated bouts of EC exercise caused an ~50% loss in TA functional capacity (measured as a reduction in Mean Isometric Torque, MIT) which is indicative of moderate muscle injury [41]. This reduction in MIT was not different among animals subsequently randomized to “No NSAID” and “NSAID” groups (No NSAID: 57.7% ± 5.7% vs NSAID: 59.8% ± 5.0%). EC injury also resulted in a marked tissue inflammatory response at 24 hrs (Fig 1) and stimulated myogenic gene expression (Fig 2). Specifically, significant early and sustained increases were observed in *myod1* (a transcription factor controlling satellite cell activation and proliferation [20]) and *vim* (a structural protein upregulated in proliferating myoblasts [42]), whereas *ptgs2* (prostaglandin synthetase 2 [aka cyclooxygenase 2, COX-2], an early mediator of muscle repair [5]), was highest at 24 hr post-injury and declined thereafter (Fig 2). As noted by others [5], expression of *ptgs1* (prostaglandin synthetase 1, [COX-1]) was only minimally increased with injury. By 48–54 hrs, *myog* (an inducer of myoblast maturation) was maximally upregulated suggesting that many proliferating myoblasts had become committed to the differentiation program.

Thus, our model fulfills multiple criteria that define acute muscle injury–namely a loss in functional capacity, changes in muscle architecture, an early influx of inflammatory cells [20] and expression of key genes involved in muscle regeneration after injury [5,23,43]. This model is therefore appropriate for the study of the effects of delayed NSAID administration on muscle regeneration after acute strain injury.

### Injury, NSAIDs and the cellular inflammatory response

Muscle regeneration depends on a highly synchronized influx of different leukocyte populations with shifting functional capacities (reviewed [44]). Specifically, after the initial neutrophil response has waned, infiltrated pro-inflammatory monocytes must switch into pro-resolution macrophages to support myogenesis [25]. To examine these dynamics in our model, total CD11b^+^ leukocytes were isolated over time from TA muscles by positive magnetic sorting. Too few cells were obtained from non-injured TA muscles to permit further analysis (not shown). In EC-injured TA muscles from untreated mice, the number of CD11b^+^ cells isolated at 24 and 48 hrs was not statistically different (Table 1); however the subpopulations were decidedly distinct. At 24 hr, neutrophils and pro-inflammatory M1 monocyte/macrophages (MO/MPs; Ly-6C^+^) predominated; few pro-resolution M2 MO/MPs (F4/80^+^) were detected. By 48 hrs, neutrophils were significantly reduced whereas the MO/MP population had increased and their phenotype was shifting from M1 to M2 as indicated by a >50% reduction in Ly-6C^+^ cells and an increase in F4/80 expression (Table 1).

**Table 1:** Dynamics and Cellular Phenotypes of Infiltrating Leukocytes after Muscle Injury.

Time Point	*N	CD11b+ cells/mg of TA muscle	Differential of CD11b+ cells (%)				Percent Ly-6C Positive MO/MP (M1)	Percent F4/80 Positive MO/MP (M2)
			MO/MP	PMNL	Eosinophils	Other^†^		
24 hr	2	3941 ± 347	42.0 ± 9.2	47.6 ± 8.8	2.1 ± 0.9	8.3 ± 4.5	42.9 ± 7.0	6.2 ± 2.7
48 hr	2	4763 ± 544	65.3 ± 9.5	14.8 ± 9.2	2.9 ± 1.6	17.0 ± 8.5	20.8 ± 3.1	16.1 ± 6.7
54 hr + saline	4	4176 ± 1204	68.0 ± 6.0	12.3 ± 5.4	7.0 ± 4.0	12.7 ± 4.5	16.8 ± 6.6	35.1 ± 5.9
54 hr + NSAID	3	3736 ± 1984	67.8 ± 10.3	13.4 ± 5.9	3.6 ± 1.7	7.8 ± 6.9	14.8 ± 5.0	28.5 ± 10.6

Animals underwent eccentric contraction-induced muscle injury as described in Methods. TA muscles were harvested at the indicated times. Tissues harvested at 54 hr were taken from animals 7 hrs after treatment with either NSAID vehicle (saline) or the non-selective NSAID, ketorolac tromethamine, given at 47 hrs post-injury. Data are means ± SD; MO/MP = Monocyte/Macrophage, PMNL = Polymorphonuclear Leukocyte.

* Each N represents TA muscles from 3 animals.

^†^Other cells include lymphocytes and other unidentified cells.

No statistical differences were observed in tissues at 54 hrs in saline control vs NSAID-treated animals.

To test whether delayed NSAID administration could alter the leukocytic inflammatory response in tissue, animals were treated 47 hrs after injury with either saline or NSAID; TA muscles were harvested 7 hrs later. At 54 hrs post-injury, vehicle-treated control animals showed a further reduction in the percent of M1 macrophages and a continued increase in M2s (Table 1) demonstrating that the initial pro-inflammatory response (PMNL, M1 macrophages) had peaked and the pro-resolution cellular response was well underway. Ketorolac administration at 47 hrs post-injury did not significantly alter the number of CD11b^+^ cells, the leukocyte differential, or the M1/M2 percentages in the tissues (Table 1).

### Injury, NSAIDs and the skeletal muscle proteome

An unbiased proteomics approach was used to investigate the global effects of injury, with and without NSAID administration, on regenerating muscles. In non-NSAID treated mice, the AMT tag proteomic approach described in Methods identified 89 significant, differentially-expressed proteins in injured muscles at 54 hrs post-EC (Table in S1 Table: Proteins Significantly Altered by Injury); of these, 67 (75%) were increased compared to non-injured TA muscles and were predominantly associated with muscle repair processes. For example, calpain (**CAN1**), a calcium-activated protease associated with the degenerative phase of muscle repair [45], was significantly increased. Proteins essential for, or indicative of, early muscle regeneration were also increased including vimentin (**VIME**), muscle-type acylphosphatase-2 (**ACYP2**), and protein unc-45 homolog B (**UN45B**). Myoblast proliferation was also suggested by significant increases in proteins driving cell division [i.e., the 55 kDa regulatory subunit of serine/threonine protein phosphatase 2A (**2ABD**) and mitochondrial fission 1 protein (**FIS1**)]. Similarly, active repair of the neuromuscular junction and the sacrolemmal membrane appeared to be underway as evidenced by increased dihydropyrimidinase-related proteins 1 and 2 (**DPYL1, 2**) and dysferlin (**DYSF**), respectively. Revascularization was also promoted via a down regulation of collagen alpha-1(XV) chain (**COFA1**) whose C-terminal region contains the anti-angiogenic factor, endostatin. Endogenous defenses against stress-induced cytotoxicity appeared activated as indicated by increases in several chaperone proteins [clusterin (**CLUS**), DnaJ homolog subfamily A member 2 (**DNJA2**), heat-shock protein beta-2 (**HSPB2**)] and an inhibitor of ROS-induced apoptosis [glutaredoxin-related protein 5 (**GLRX5**) [46]]. Serum-associated proteins such as albumin (**ALBU**) and serotransferrin (**TRFE**) were also significantly increased indicating that edema remains a prominent feature at this point in the injury resolution process.

**Fig 3:**
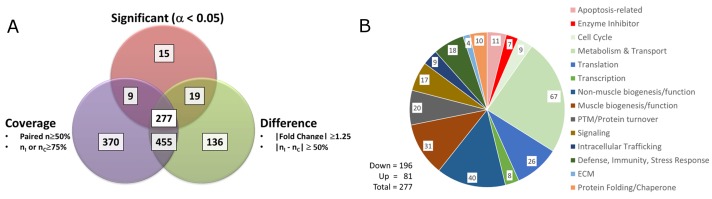
AMT-tag proteomics identifies proteins from injured muscles that are significantly altered by NSAID treatment. (A) Proteins that met both the criteria of “Coverage” (defined as: [Number of paired injured and control muscles ≥ 50% or (N_(injured)_ or N_(control)_) ≥75%]) and of “Difference” (defined as: [|Fold Change| ≥1.25 or |N_(injured)_—N_(control)_| ≥ 50%)]) were subjected to statistical analysis to identify those that were significantly affected by NSAID treatment. Proteins were analyzed for quantitative effects using a standard T-test and for qualitative effects using G-test methodology [32]. In total, 277 differentially expressed proteins were found to be significantly altered by NSAID administration. (B) Functional categories of the 277 differentially expressed proteins (assigned as described in Methods; Table in S2 Table: Proteins Significantly Altered by NSAID) are depicted. PTM: post-translational modification; ECM: extracellular matrix.

In contrast, NSAID administration significantly altered 277 proteins in the injured TA muscles (Fig 3A; also see Table in S2 Table: Proteins Significantly Altered by NSAID) compared to injured muscles from vehicle-treated animals. Of these, 71% (196/277) were decreased by NSAIDs including 14 metabolism and pro-biogenesis proteins that were found to be significantly increased by injury alone (Table 2 and S2 Table: Proteins Significantly Altered by NSAID). These findings support a direct NSAID-induced reversal of key repair responses to injury.

**Table 2:** Common proteins significantly altered by injury and by NSAID administration.

ID	Protein Name	Accession Number	Functional Category	Function	Response to Injury Alone	Response to Injury + NSAIDs
GCP2	Gamma-tubulin complex component 2	Q921G8	Cell cycle control	Involved in microtubule nucleation at the centrosome	down	up
ATLA2	Atlastin-2	Q6PA06	Non-muscle cell specific biogenesis	GTPase that functions in endoplasmic reticulum tubular network biogenesis	down	up
GLRX5	Glutaredoxin-related protein 5; mitochondrial	Q80Y14	Apoptosis-related	Involved in iron homeostasis; protects against ROS-induced apoptosis	up	down
FIS1	Mitochondrial fission 1 protein	Q9CQ92	Cell cycle control	Involved in mitochondrial fission during cell replication	up	down
K2C7	Keratin; type II cytoskeletal 7	Q9DCV7	Cell cycle control	Blocks interferon-dependent interphase and stimulates DNA synthesis	up	down
ARFG1	ADP-ribosylation factor GTPase-activating protein 1	Q9EPJ9	Intracellular trafficking	Involved in membrane trafficking and vesicle transport	up	down
ALBU	Serum albumin	P07724	Metabolism & Transport	Main protein of plasma	up	down
EST1C	Carboxylesterase 1C	P23953	Metabolism & Transport	A carboxylesterase	up	down
ESTD	S-formylglutathione hydrolase	Q9R0P3	Metabolism & Transport	Thioester hydrolase	up	down
ACYP2	Acylphosphatase-2; muscle type isozyme	P56375	Muscle cell specific biogenesis	Acylphosphatase targeting the Ca2+/Mg2+-ATPase from sarcoplasmic reticulum of skeletal muscle; increased with muscle differentiation	up	down
FHL3	Four and a half LIM domains protein 3	Q9R059	Muscle cell specific biogenesis	A novel alpha7/beta1 integrin-interacting protein	up	down
DPYL2	Dihydropyrimidinase-related protein 2	O08553	Non-muscle cell specific biogenesis	Plays a role in neuronal development and polarity	up	down
K2C8	Keratin; type II cytoskeletal 8	P11679	Non-muscle cell specific biogenesis	Helps link the contractile apparatus to dystrophin at muscle costameres	up	down
MOES	Moesin	P26041	Non-muscle cell specific biogenesis	Probably connect major cytoskeletal structures to the plasma membrane	up	down
ACBP	Acyl-CoA-binding protein	P31786	Signal transduction	Intracellular carrier of acyl-CoA esters	up	down
DYSF	Dysferlin	Q9ESD7	Signal transduction	Key Ca++ sensor involved in sarcolemma repair after mechanical stress injury	up	down
CES1D	Carboxylesterase 1D	Q8VCT4	Metabolism & Transport	Major lipase in white adipose tissue	up	up
EST1	Liver carboxylesterase 1	Q8VCC2	Metabolism & Transport	Liver detoxification enzyme.	up	up
CYTB	Cystatin-B	Q62426	Non-muscle cell specific biogenesis	An intracellular thiol proteinase	up	up
DNJA2	DnaJ homolog subfamily A member 2	Q9QYJ0	Protein folding/chaperone	Co-chaperone of Hsc70	up	up
ELOC	Transcription elongation factor B polypeptide 1	P83940	Transcription	Aka elongin; a general transcription elongation factor	up	up
HNRPC	Heterogeneous nuclear ribonucleoproteins C1/C2	Q9Z204	Translation	Binds pre-mRNA and nucleates the assembly of 40S hnRNP particles	up	up

Comparison of protein profiles in “Non-Injured vs Injured TA muscles in vehicle-treated mice” and in “TA muscles from injured mice treated with or without NSAID” revealed 22 shared proteins that were significantly altered in both groups. Most proteins (91%) were significantly increased by injury and were involved in metabolism, biogenesis and survival. In 16 of 22 cases (73%), NSAID administration reversed the injury-induced response.

**Fig 4:**
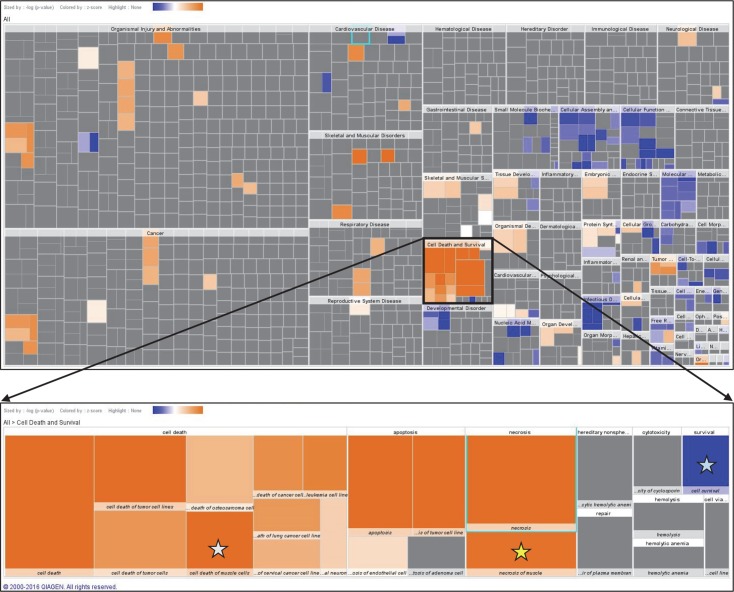
IPA reveals “Cell Death and Survival” as the major biofunction affected by NSAID administration after muscle injury. Heat maps of biological functions in which significantly altered proteins were associated with NSAID administration. Color scale is based on z-score with orange indicating predicted activation and blue indicating inhibition. ***Top***: Global view of all returned biofunctions. ***Bottom***: Enlarged view of “Cell Death and Survival” showing “Cell Death of Muscle Cells” (white star), “Necrosis of Muscle” (yellow star) and “Cell Survival” (blue star).

IPA core analysis of the 277 NSAID-regulated proteins returned “Cell Death and Survival” as the top molecular and cellular functional category (128 unique proteins; *P* < .001; Fig 4). The activation state of all subcategories involving cell death was predicted to be increased, whereas those involving cell survival and proliferation were predicted to be decreased (Fig 4). Interestingly, 19 proteins mapped to subcategories of “Necrosis of Muscle” and/or to “Cell Death of Muscle”. These subcategories had z-scores of 2.729 and 3.085, respectively, predicting an increased activation state (Table 3). In contrast, the subcategories of “Cell Survival” (43 proteins; z-score = -3.339) and “Cell Proliferation” (103 proteins; z-score = -2.721) were predicted to be decreased by NSAID administration (Table 3).

**Table 3:** IPA predicts an NSAID-induced Increase in Cell Death and a Reduction in Proliferation/Survival in Regenerating TA Muscles.

Diseases or Functions Annotation	p-Value	Predicted Activation State	Activation z-score	# Molecules	Molecules
Necrosis of Muscle	2.15E-06	Increased	2.729	19	ALDOA, APIP, BAG1, CACNB2, CALR, COL6A1, CYCS, DYSF, EEF1A2, EEF1D, HK1, HSPB6, HSPE1, MDH1, MYH4, MYH6, RAF1, TNNT1, TPT1
Cell Death of Muscle	2.30E-05	Increased	3.085	17	ALDOA, APIP, BAG1, CACNB2, CALR, CYCS, DYSF, EEF1A2, EEF1D, HK1, HSPB6, HSPE1, MDH1, MYH4, RAF1, TNNT1, TPT1
Proliferation of Cells	6.72E-07	Decreased	-2.721	103	ACTN1, AHCY, AIMP1, AKR1B1, AKR1B10, ALB, ALDOA, ANXA1, ANXA2, ANXA6, ARAF, BAG1, BIN1, BRAF, C3, CACNA1D, CACNA1S, CACNB3, CALR, CAPZA1, CCT2, CFL1, CNBP, CNPY2, COL4A2, COL6A1, COL6A2, CSNK1D, CSRP3, CTSD, DBI, DES, DLST, DNAJA2, DPYSL2, EEF1D, EIF5A, EIF5A2, EML1, ETFB, EZR, F2, FBN2, FHL1, FIS1, GPX3, HK1, HNRNPC, HNRNPD, HSPA4, HSPA5, HSPB6, ITGB1, KRT7, KRT8, LAMB2, LAP3, MYH10, MYH6, MYH7, NACA, NDRG2, NME1, NUDCD3, PDIA3, PEBP1, PKM, PPIA, PPIB, PPP1CA, PPP1CB, PPP2CA, PRDX1, PRPH, PSMC4, PTGES3, RAF1, RPS14, RPS19, SERPINA1, SERPINA3, Serpina3g (includes others), SERPINC1, SPEG, SPTAN1, TAGLN, TAGLN2, TF, Tmsb4x (includes others), TPT1, TTR, TUBB, TUBB2A, TUBB3, TUBB4B, TXN2, TXNDC5, UBE2L3, USMG5, VCP, VIM, YBX3, YWHAZ
Cell Survival	2.08E-04	Decreased	-3.339	43	AIMP1, ALB, BAG1, BRAF, C3, CACNB3, CALR, EEF2, EIF2S1, EZR, F2, GLUD1, HK3, HSPA4, HSPA5, HSPB6, ITGB1, KHK, NDRG2, NME1, PDIA3, PKLR, PKM, PPIA, PPIB, PPP1CA, PPP1CB, PPP2CA, PRDX6, PRPH, PSMC4, RAF1, RPL38, TPT1, TUBB, TUBB3, TUBB4A, TXNDC5, UBE2L3, USMG5, VCP, VIM, YWHAZ

Closer inspection by Gene Ontology analysis and available information in the NCBI protein database showed that the proteins affected by NSAID administration are broadly distributed and enriched in the nucleus, mitochondria, neuromuscular junction and plasma membrane. These proteins are involved in several key biological functions (Fig 3B; also see Table in S2 Table: Proteins Significantly Altered by NSAID) including muscle metabolism, biogenesis and function, and cellular processes including apoptosis as described below.

### Metabolism and transport

This functional category contained the greatest number of NSAID-affected proteins (68/277; 24.5%). While not muscle-specific, these proteins are heavily involved in muscle energetics and 88% were down-regulated by NSAIDs including those involved in glycolysis, TCA cycle, electron transport and beta-oxidation. This finding is consistent with the known ability of NSAIDs to delay muscle regeneration after injury [6,8,10] and to suppress muscle metabolism after strength-training exercise (reviewed in [47]).

### Muscle specific biogenesis/structure/function

*Myogenesis*: NSAID administration negatively impacted several proteins associated with post-injury myogenesis. Loss of desmin (**DESM**) is a hall-mark feature of muscle injury [48] and NSAID treatment significantly reduced this protein. Vimentin (**VIME**), an intermediate filament protein expressed in regenerating but not mature muscle fibers [49,50] was also down-regulated. Although vimentin is expressed by other cell types including macrophages[51], results from our cellular studies above suggest that the NSAID-induced reduction in vimentin was not due to reduced inflammatory cell influx. Proteins that regulate myoblast differentiation/maturation were also negatively affected by NSAID administration including ankrin repeat domain-containing protein-2 (**ANKR2,** a coordinator of proliferation and apoptosis during myoblast differentiation; also abbreviated ANKRD2) [52], cysteine and glycine-rich protein 3 (**CSRP3,** also known as muscle LIM protein [MLP]**–**a critical positive regulator of myogenic differentiation [53]) and nascent polypeptide-associated complex subunit alpha; muscle-specific form (**NACAM**—a muscle-specific transcription factor present in differentiated myotubes but not myoblasts). In contrast, injury alone increased ANKR2 and CSRP3 by 2.5 and 2.1-fold, respectively. Although these latter increases did not reach statistical significance (p = .07), they are consistent with studies by Barash et al whose genomic profiling of EC-injured mouse TA muscles showed significant increases in ANKRD2 and CSRP3/MLP gene expression that peaked at ~12 hrs post-injury and remained elevated at 48 hrs [23].

*Sarcolemmal Repair*: EC-induced injury is associated with disruptions in sarcolemmal integrity and repair of such lesions depends on the coordinated interaction of tripartite motif-containing protein 72 (**TRI72**), dysferlin (**DYSF**) [54,55] and microtubules [56]. Our data demonstrate that NSAID administration reduced TRI72, DYSF and multiple members of the beta tubulin family (**TBB2A, 2B, 4A, 4B, 6**). To our knowledge, these data provide the first evidence that NSAIDs may interfere with repair of sarcolemmal integrity after injury.

*Cytoskeleton*: NSAID administration also significantly impacted multiple myocytoskeletal proteins. Specifically, 6 isoforms of adult skeletal muscle myosin heavy chain (MYH), 1 perinatal myosin heavy chain and 1 myosin regulatory light chain were significantly increased by NSAID administration. Three of these (**MYH4, MYH6**, and **MRLS**) are characteristic of fast-twitch muscles, such as the TA muscle used here, whereas **MYH1, 7** and **7B** are largely associated with slow-twitch fibers. Actin bundling/capping proteins were also increased including F-actin-capping protein subunits alpha-1 and -2 (**CAZA1, CAZA2**), cofilin-1 (**COF1**), and two forms of transgelin (**TAGL, TAGL2**). NSAID administration also significantly increased 3 members of the actinin family (**ACTN1, 2, 3**), myozenin-3 (**MYOZ3**) and telethonin (**TELT**). ACTN2 and -3 are skeletal muscle-specific and, together with MYOZ3 and TELT, link Z-line proteins to the myofibrillar actin filaments of the sarcomere.

In contrast, other Z-line associated proteins that link the sarcomere to the costamere [57] were decreased by NSAID treatment including DESM and CSRP3/MLP (mentioned above), zinc finger domain-containing proteins known as four-and-a-half LIM domain proteins 1 and 3 (**FHL1, 3**), PDZ and LIM domain protein 3 (**PDLI3)**, and xin actin-binding repeat-containing protein 2 (**XIRP2).**

### Apoptosis

Several anti-apoptosis proteins were significantly down-regulated by NSAID administration, including two members of the 14-3-3 family (**1433E**, **1433Z**; [58]), translationally-controlled tumor protein (**TCTP**) [59], the mitochondrial proteins 3-ketoacyl-CoA thiolase (**THIM;** aka acetyl-Coenzyme A acyltransferase; [60]) and thioredoxin (**THIOM;** [61]), mitochondrial fission process protein 1 (**MTFP1;** aka mitochondrial 18kDa protein; [62]), and methylthioribulose-1-phosphate dehydratase (**MTNB**)–an inhibitor of the caspase 9/cytochrome c/APAF-1-dependent cell death pathway [63]. Calreticulin (**CALR**) was also down-regulated; cell-surface CALR serves as a general recognition ligand on apoptotic cells that facilitates their recognition and removal during resolution of inflammation [64]. Although only represented by a single peptide in our dataset, the novel anti-apoptosis protein Bcl-2-associated athanogene-1 (**BAG1**) was highly down-regulated by NSAID administration (>13-fold reduction; p = 0.002).

**Fig 5:**
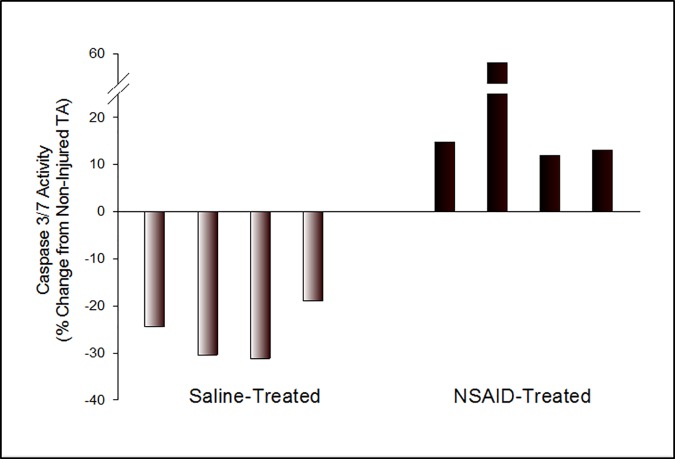
NSAID-induced decrease in anti-apoptosis proteins is associated with increased caspase activity. Caspase 3/7activity in muscle homogenates from saline- and NSAID-treated animals (4/group) was measured at 54 hrs after injury (7 hrs post-treatment) by commercial ELISA. Data are given as the fold-change for each animal relative to its own non-injured TA muscle. Responses between saline versus NSAID-treated animals were statistically significant as determined by ANOVA using a mixed model with random effect of animal within treatment with the level of significance set at p < .05.

The NSAID-induced decrease in anti-apoptosis proteins was associated with a significant increase in caspase 3/7 enzymatic activity in injured TA muscle homogenates from 4 of 4 ketorolac-treated animals compared to non-injured muscle (mean increase = 23.7%, p = .02, Fig 5). In contrast, caspase 3/7 activity in injured TA muscles from saline-treated animals was significantly reduced in all animals tested (mean decrease = 26.2%; p = .005, Fig 5).

## Discussion

Antecedent non-penetrating soft tissue injury often precedes the development of severe bacterial infection, including the ~50% of patients with group A streptococcal necrotizing fasciitis/myonecrosis who lack an obvious portal of bacterial entry (reviewed in [65]). Although some epidemiologic factors that increase the risk of death have been defined, the risk factors for initiation of infection are less clear. Non-penetrating trauma and use of non-steroidal anti-inflammatory drugs (NSAIDs) have each been associated with infection onset and/or worse outcomes in humans and in animal models of this infection (reviewed in [16]). For instance, in 2007, a retrospective study found that patients with GAS necrotizing fasciitis (but not those with GAS cellulitis) were 6 times more likely than matched controls to have a recent history of blunt trauma [66]. Further, a 2008 prospective epidemiologic study showed that NSAID use was independently associated with increased risk for severe GAS infection [67]. Our own work has shown that NSAID administration promotes trafficking of circulating GAS to sites of injury [18], greatly accelerates progression of established infection and reduces antibiotic efficacy [19].

To investigate how NSAIDs might predispose injured muscle to infection, the present study exploited our established animal model of eccentric contraction (EC)-induced muscle injury. Unlike previous reports in which NSAIDs were administered prior to experimental injury or excessive exercise, the present studies were designed to more closely mimic the human scenario in which NSAIDs are typically used during the peak of perceived muscle soreness–typically 24–48 hrs post-injury. At this point post-injury, markers of inflammation and muscle dysfunction are objectively measurable. For example, Lieber’s group has shown in mouse and rat models that muscle force-generating capacity remains significantly reduced at 24–48 hrs following experimental strain-associated muscle injury [23,24] and that this dysfunction is associated with marked architectural and inflammatory changes and in myoregulatory gene expression [23,24]. Results of the genetic, histologic and functional studies presented here are consistent with these reports and provide evidence that active muscle regeneration is ongoing at the time chosen for NSAID administration (47 hr post-injury) in our model.

Also within this critical 24–48 hr timeframe after injury, Arnold and colleagues have shown that infiltrated pro-inflammatory M1 macrophages convert to an anti-inflammatory/pro-resolution M2 phenotype to sustain myogenic differentiation, myofiber growth and membrane repair [25]. Because such phenotype switching is critical for muscle regeneration after injury and because prostaglandin metabolites (e.g. 15d- PGJ_2_) promote the M2 phenotype [68], we first investigated whether delayed NSAID use might directly affect the M1 to M2 transition. Ketorolac, given after the peak of M1 influx, had no significant effect on the numbers or functional phenotypes of macrophages in the injured TA muscles. These data suggest that the signaling events promoting macrophage phenotype switching either occurred prior to NSAID administration or are NSAID-insensitive. Further, these results suggest that any NSAID-induced deficit in functional muscle regeneration is not related to effects on pro-resolution M2 leukocyte dynamics at this NSAID administration time point.

Like macrophages, muscle fibers can alter their functional phenotype under certain conditions such as changes in nerve supply, loading/unloading and aging [69]. Our findings suggest that NSAID use should be included on this list. Specifically, we demonstrate a NSAID-associated increase in slow MYH isoforms in the fast-twitch TA muscle which could, in part, explain the purported loss of muscle strength associated with NSAID use. The mechanism responsible is not apparent here, though the observed reduction in the thyroid hormone transport protein, transthyretin (TTHY; 3.19-fold decrease) could play a role [70] Further, by increasing MYOZ3, NSAID administration could activate the calcineurin/NFAT signaling pathway that drives transcription of slow fiber type-specific genes even in fast-twitch muscles [69]. An NSAID-induced transition of muscle fiber type could have clinical import in children, in those lacking normal muscle regenerative capacity, in patients with progressive loss of muscle mass (elderly, AIDS), and those undergoing rehabilitation after trauma or surgery. More research is required to confirm this notion.

Muscle repair/regeneration also requires repair of the saracolemmal membrane, revascularization and re-innervation of the myofiber, and reconstitution of the extracellular matrix [71]. Our proteomics data suggest that at 48–54 hrs after EC injury, muscle repair is well underway and is dominated by proliferation of muscle cell precursors and upregulation of stress-response/damage control mechanisms. Our data further suggest a damage-control, pro-survival process is also engaged. Each of these restorative processes was deleteriously affected by NSAID administration.

The NSAID-induced down-regulation of multiple inhibitors of the mitochondrial-based intrinsic apoptosis pathway was particularly intriguing. The 14-3-3 family of proteins, via binding to pro-apoptotic moieties such as Bcl-2-associated death promoter (BAD) and other ligands, is critical for cell survival signaling [58]. TCTP prevents apoptosis by inserting into the mitochondrial membrane and blocking Bax dimerization [72,73] and may also destabilize the tumor suppressor (pro-apoptosis) protein p53 [74,75]. Translational regulation of TCTP occurs via the PI3K/Akt/mTORC1 pathway [76]. Zhang and colleagues have recently shown that mTOR is essential for myogenesis [77]. Thus, our data may supply a missing link in the pathway between injury and regeneration and suggest that an injury/TCTP/mTOR axis drives this process. MTNB (also known as Apaf-1-interacting protein, APIP [78]) was initially identified by Cho et al as an inhibitor of hypoxia-induced intrinsic apoptosis in skeletal muscle [63] where it competes with caspase 9 for binding to Apaf-1 of the active apoptosome. APIP/MTNB also inhibits cytochrome c-induced activation of caspase-9 [63,79]. These activities are independent of its enzymatic function in methionine salvage [80]. Lastly, BAG1 was significantly reduced by NSAIDs. BAG1 is a novel multifunctional protein that was first identified via its ability to functionally augment the anti-apoptotic protein, Bcl-2 [81]. Recently, Warren et al demonstrated that contraction-induced injury, but not freeze injury, induced high level gene expression of a related protein, BAG3 [82]. In our study, the functional consequence of NSAID-induced down-regulation of these anti-apoptosis proteins was increased caspase activity in the injured muscles.

Interestingly, studies have suggested that controlled apoptosis is required for myogenic differentiation [83,84] and Fernando et al have implicated activated caspase-3 in this process. In their studies, caspase-3 activity was maximally (but transiently) increased in cultured murine C2C12 myoblasts after 24 hrs of serum withdrawal-induced differentiation. Biochemical blockade or genetic ablation of caspase-3 dramatically reduced myotube formation. Other data from this study suggested that the differentiation-associated caspase-3 activity was not strictly apoptotic since traditional markers of apoptosis (i.e., PARP cleavage, Annexin V staining) were absent or unchanged in these cells. In contrast to their findings, our data demonstrate an overall down-regulation of caspase activity in muscles undergoing regeneration at 54 hrs after strain injury (Fig 5, left). Further, our immunohistologic studies and western blot analysis of injured vs non-injured TA muscles from untreated mice failed to demonstrate an injury-induced increase in activated caspase-3 at this time (not shown). These apparently dichotomous results might be explained by differences in the model systems used (i.e., *in vitro* cultured cells with serum starvation vs whole muscles undergoing regeneration after strain injury) or the single time of sampling in our *in vivo* model (i.e., 54 hr post injury). We also demonstrate here that NSAID administration significantly increased caspase activity in regenerating muscles (Fig 5, right). Here too, we found no evidence of caspase-3 activation in tissues or tissue homogenates from NSAID-treated mice (not shown). In the absence of detectable caspase-3 activity and because our enzymatic detection assay measures both active caspase-3 and -7, it suggests a possible role for caspase-7 in the NSAID-induced response. Further, it is also conceivable that some activity attributed by Fernando et al to caspase-3 may in fact be related to caspase-7 since *1)* these enzymes share overlapping substrate specificities [85] and *2)* because the pharmacologic inhibitor used by these investigators (Z-DEVD.fmk) also inhibits caspase-7 [86]. Caspase-7 is an endoplasmic reticulum (ER)-associated caspase and can be regulated by the ER stress response protein known as glucose-regulated protein 78 (GRP78) [87] (also shown here to be reduced by NSAID administration). Thus, we hypothesize that GRP78-based inhibition of caspase-7 activity is required to ensure muscle regeneration after strain-induced injury and that NSAIDs delay repair by relieving this inhibition. Elucidation of this potential mechanism this requires further study.

## Conclusions

In summary, our data provide new evidence suggesting that NSAID use during the peak of post-injury pain and inflammation decreases muscle metabolism, prevents neuromuscular junction and sarcolemmal repair, stimulates muscle fiber type transition and promotes a pro-apoptosis phenotype. Such findings provide molecular evidence supporting the notion that NSAIDs have a negative influence on myogenesis, including reducing muscle strength post-healing. By promoting cell death, NSAIDs could expand the nidus of injury, thereby increasing muscle susceptibility to post-injury infection [18,19]. These findings support renewed concerns about the risks versus benefits of NSAID use, especially in those with increased susceptibility to contraction-induced injury, those with muscle wasting or poor regenerative capacity, and those at risk for life-threatening bacterial myonecrosis. Given the potential to change the current paradigm of pain management in these settings, studies to validate these results in humans are warranted and must include NSAIDs of different classes given at different times post-injury.

## Supporting information

S1 Table Proteins Significantly Altered by Injury. (XLSX)

S2 Table Proteins Significantly Altered by NSAID. (XLSX)

S1 Supporting Information Additional Methodology for Proteomics Analysis. (DOCX)
