# Supplementary material for: Effects of delayed NSAID administration after experimental eccentric contraction injury – A cellular and proteomics study
Source: PLoS One. 2017 Feb 28;12(2):e0172486. doi: 10.1371/journal.pone.0172486 (PMC5330483; doi:10.1371/journal.pone.0172486)
Supplement: S1 Supporting Information — (DOCX) [file pone.0172486.s003.docx]

**Detailed Proteomics Methods**

Chemicals

All chemicals and reagents were purchased from Sigma-Aldrich (St. Louis, MO) unless stated otherwise. Sequencing-grade modified trypsin was purchased from Affymetrix (Santa Clara, CA), and bicinchoninic acid (BCA) assay reagents and standards were obtained from Thermo Scientific (Rockford, IL). Purified, deionized water, >18 MΩ, (Nanopure Infinity ultrapure water system, Barnstead, Dubuque, IA) was used to make all aqueous buffers.

Whole Muscle Protein Extraction and Digestion

All sample preparations were performed in a dedicated biosafety cabinet according to institutional guidelines until muscle proteins were proteolytically cleaved to peptides, at which point samples were considered non-infectious. Samples were blocked and randomized according to treatment to minimize the effects of systematic biases and to ensure the even distribution of known and unknown confounding factors across the entire experimental [1].

Muscle tissues were placed into individual 2 mL snap-cap tubes (Eppendorf, Hamburg, Germany) with 400 µL of 100 mM ammonium bicarbonate, pH 8, and a 3 mm stainless steel bead. The tissues were then homogenized using a TissueLyser II system (Qiagen, Valencia, CA) at 30 Hz for 2 min on each side. A portion of each homogenate (200 µL) was removed and placed into a fresh tube containing 156 mg of urea (for a final concentration of 8 M) and vortexed into solution (the remaining homogenates were flash frozen in liquid nitrogen and stored at -80°C). Protein concentrations were determined with a BCA protein assay. Proteins were reduced with 10 mM dithiothreitol (DTT), followed by alkylation of free sulfhydryl groups with 40 mM iodoacetamide in the dark; each reaction was performed for 1 h at 37°C with constant shaking at 800 rpm. Denatured and reduced samples were diluted 10-fold with 50 mM ammonium bicarbonate pH 7.8, and CaCl_2_ was added to a final concentration of 1 mM prior to enzymatic digestion. Sequencing-grade modified trypsin was activated by incubating for 10 min at 37˚C. Activated trypsin was then added to the samples at 1:50 (w/w) trypsin-to-protein ratio, and samples were digested at 37˚C for 3 h with constant shaking at 800 rpm; reactions were quenched by rapid freezing in liquid nitrogen. Digested samples were desalted using a 4-probe positive pressure Gilson GX-274 ASPEC™ system (Gilson Inc., Middleton, WI) with Discovery C18 100 mg/1 mL solid phase extraction tubes (Supelco, St.Louis, MO), using the following protocol: 3 mL of methanol followed by 2 mL of 0.1% TFA in H_2_O were added for column conditioning; samples were then loaded onto each column followed by a column wash with 4 mL of 95:5: H_2_O:ACN, 0.1% TFA; finally, samples were eluted with 1 mL 80:20 ACN:H_2_O, 0.1% TFA. The samples were then dried down to ~30 µL *in vacuo,* and a final BCA assay was performed to determine peptide concentrations. The samples were then vialed for LC-MS/MS analysis.

Fractionation of Protein Digests

To provide comprehensive coverage of the mouse muscle proteome, aliquots of individual sample protein digests were pooled and subjected to high pH reversed-phase liquid chromatography fractionation [2]. Equal amounts of protein mass from each sample were pooled into one tube to a final mass of 300 µg and diluted to 900 µL with 10 mM ammonium formate (pH 10.0). Peptides were separated using an XBridge C18 column (250 x 4.6 mm, 5 μm) equipped with a 4.6 x 20 mm guard column of the same material (Waters, Milford, MA). Separations were performed at 0.5 mL/min using an Agilent 1100 series HPLC system (Agilent Technologies, Santa Clara, CA) with mobile phases (A) 10 mM ammonium formate, pH 10, and (B) 10 mM ammonium formate, pH 10.0/acetonitrile (10:90). The gradient was as follows: 0%-5% B, 0-10 min; 5%-35% B, 10-70 min; 35%-70% B, 70-85 min; hold at 70% B, 85-95 min; 70%-0% B, 95-105 min; hold at 0% B, 105-120 min. Fractions were collected into a 96-well plate every 1.25 min (96 fractions over the entire gradient). The plate was partially dried *in vacuo,* and every 24th fraction was combined to yield 24 total fractions; to increase recovery of peptides, each well was rinsed with 50% acetonitrile, and the rinse was added to the appropriate sample. The fractions were then completely dried *in vacuo* and reconstituted in 25 µL of 25 mM ammonium bicarbonate prior to storage at -20°C until LC-MS/MS analysis.

Reversed-phase capillary LC-MS/MS and LC-MS analyses

LC-MS/MS analysis was used to identify peptide sequences for generating an accurate mass and time (AMT) tag database for mouse muscle homogenates (see below). For this, each fraction was analyzed using a 2-column custom-built capillary LC system. The reversed-phase columns were prepared in-house by slurry packing 3-µm Jupiter C_18_ particles (Phenomenex, Torrence, CA) into 35-cm x 360 µm o.d. x 75 µm i.d fused silica capillaries (Polymicro Technologies Inc., Phoenix, AZ) using a 1-cm sol-gel frit for media retention [3]. Mobile phases consisted of 0.1% formic acid in water (A) and 0.1% formic acid in acetonitrile (B). The flow rate was 300 nL/min with a gradient profile as follows: 0% B, 0-40 min; 0-8% B, 40-42 min; 8-12% B, 42-60 min; 12-35% B, 60-115 min; 35-60% B, 115-137 min; 60%-100% B, 137-140 min. The LC system was coupled online to a hybrid linear ion trap-Orbitrap mass spectrometer (Orbitrap Velos; Thermo Scientific, San Jose, CA) by way of an in-house manufactured electrospray ionization interface. The temperature of the heated capillary and the ESI voltage were 350°C and 2.2 kV, respectively. Data were acquired for 100 min, beginning 40 min after sample injection. Orbitrap spectra (AGC 1x10^6^) were collected from 400-2000 *m/z* at a resolution of 60k followed by data dependent acquisition of MS/MS spectra (AGC 1x10^4^) of the ten most abundant ions using a 2 amu isolation width and 35% collision energy. A dynamic exclusion time of 45 s was used to discriminate against previously analyzed ions (within -0.55 and 1.55 amu).

Following AMT tag database generation, LC-MS analyses were performed on all individual muscle homogenate samples to generate quantitative proteomics data. For this, each sample was analyzed in random order using identical chromatographic and electrospray conditions as for LC-MS/MS analyses. The LC system was interfaced to an LTQ-Orbitrap Velos mass spectrometer (Thermo Scientific), and Orbitrap spectra were collected over the mass range 400-2,000 m/z.

Population of the AMT tag database

As described above, TA muscles from animals in the different experimental groups were homogenized, the associated proteins subjected to proteolytic digestion, and aliquots of protein digests pooled. The pooled digests were then subjected to offline fractionation, followed by analysis of each fraction using LC-MS/MS. The resulting instrument data files were then processed using the MS-Generating Function (MS-GF) [4] with matching to the mouse and *Streptococcus pyogenes* protein databases (Uniprot), in order to identify peptides from tandem mass spectra. The following post-translational modifications were considered: static iodoacetylation of cysteine residues (+57.0215 Da); dynamic oxidation of methionine residues (+15.9949 Da); dynamic deamidation of arginine residues (+0.9840 Da); and dynamic ADP ribosylation of arginine residues (+541.0611 Da). To increase the accuracy of the assignment of parent ion masses, LC-MS/MS data were processed sequentially with DeconMSn [5] and DTARefinery [6] prior to MS-GF processing, which has been shown to increase the number of peptide spectral matches at a given false discovery rate. The peptide identifications were then filtered using a MS-GF spectral probability of ≤1x10^-10^ and a parent ion mass tolerance of ±4 ppm, which resulted in 58,484 filter passing unique peptides at a peptide level false discovery rate of <0.1%, based on a target-decoy database search [7]. The sequences of these peptides, their calculated monoisotopic masses, and normalized elution times (NETs) were then used to populate an accurate mass and time (AMT) tag library. .

Processing of Quantitative LC-MS Datasets

Quantitative LC-MS datasets were processed using the PRISM Data Analysis system [8], which is a series of software tools developed in-house (e.g. Decon2LS [9] and VIPER [10] freely available at omics.pnl.gov/software). Individual steps in this data processing approach are reviewed here [11]. The peptide identities of detected features in each dataset (here a dataset is equivalent to a single LC-MS analysis) were determined by comparing their measured monoisotopic masses and NETs to the calculated monoisotopic masses and observed NETs of each of the peptides in the filtered AMT tag databases within initial search tolerances of ± 6 ppm and ± 0.025 NET for monoisotopic mass and elution time, respectively. The peptides identified from this matching process and the associated integrated LC-MS peak areas were retained as a matrix for subsequent data analysis.

Reference List

1. Oberg AL, Vitek O (2009) Statistical design of quantitative mass spectrometry-based proteomic experiments. J Proteome Res 8: 2144-2156.

2. Yang F, Shen Y, Camp DG, Smith RD (2012) High-pH reversed-phase chromatography with fraction concatenation for 2D proteomic analysis. Expert Rev Proteomics 9: 129-134.

3. Maiolica A, Borsotti D, Rappsilber J (2005) Self-made frits for nanoscale columns in proteomics. Proteomics 5: 3847-3850.

4. Kim S, Mischerikow N, Bandeira N, Navarro JD, Wich L, Mohammed S, Heck AJ, Pevzner PA (2010) The generating function of CID, ETD, and CID/ETD pairs of tandem mass spectra: applications to database search. Mol Cell Proteomics 9: 2840-2852.

5. Mayampurath AM, Jaitly N, Purvine SO, Monroe ME, Auberry KJ, Adkins JN, Smith RD (2008) DeconMSn: a software tool for accurate parent ion monoisotopic mass determination for tandem mass spectra. Bioinformatics 24: 1021-1023.

6. Petyuk VA, Mayampurath AM, Monroe ME, Polpitiya AD, Purvine SO, Anderson GA, Camp DG, Smith RD (2010) DtaRefinery, a software tool for elimination of systematic errors from parent ion mass measurements in tandem mass spectra data sets. Mol Cell Proteomics 9: 486-496.

7. Elias JE, Gygi SP (2010) Target-decoy search strategy for mass spectrometry-based proteomics. Methods Mol Biol 604: 55-71.

8. Kiebel GR, Auberry KJ, Jaitly N, Clark DA, Monroe ME, Peterson ES, Tolic N, Anderson GA, Smith RD (2006) PRISM: a data management system for high-throughput proteomics. Proteomics 6: 1783-1790.

9. Jaitly N, Mayampurath A, Littlefield K, Adkins JN, Anderson GA, Smith RD (2009) Decon2LS: An open-source software package for automated processing and visualization of high resolution mass spectrometry data. BMC Bioinformatics 10: 87.

10. Monroe ME, Tolic N, Jaitly N, Shaw JL, Adkins JN, Smith RD (2007) VIPER: an advanced software package to support high-throughput LC-MS peptide identification. Bioinformatics 23: 2021-2023.

11. Zimmer JS, Monroe ME, Qian WJ, Smith RD (2006) Advances in proteomics data analysis and display using an accurate mass and time tag approach. Mass Spectrom Rev 25: 450-482.
